# Diving into the zebrafish brain: exploring neuroscience frontiers with genetic tools, imaging techniques, and behavioral insights

**DOI:** 10.3389/fnmol.2024.1358844

**Published:** 2024-03-12

**Authors:** O. Doszyn, T. Dulski, J. Zmorzynska

**Affiliations:** Laboratory of Molecular and Cellular Neurobiology, International Institute of Molecular and Cell Biology in Warsaw (IIMCB), Warsaw, Poland

**Keywords:** modern methods for neuroscience, brain development, genetic tools, behavioral studies, optogenetics, brain imaging, virtual reality, zebrafish

## Abstract

The zebrafish (*Danio rerio*) is increasingly used in neuroscience research. Zebrafish are relatively easy to maintain, and their high fecundity makes them suitable for high-throughput experiments. Their small, transparent embryos and larvae allow for easy microscopic imaging of the developing brain. Zebrafish also share a high degree of genetic similarity with humans, and are amenable to genetic manipulation techniques, such as gene knockdown, knockout, or knock-in, which allows researchers to study the role of specific genes relevant to human brain development, function, and disease. Zebrafish can also serve as a model for behavioral studies, including locomotion, learning, and social interactions. In this review, we present state-of-the-art methods to study the brain function in zebrafish, including genetic tools for labeling single neurons and neuronal circuits, live imaging of neural activity, synaptic dynamics and protein interactions in the zebrafish brain, optogenetic manipulation, and the use of virtual reality technology for behavioral testing. We highlight the potential of zebrafish for neuroscience research, especially regarding brain development, neuronal circuits, and genetic-based disorders and discuss its certain limitations as a model.

## 1 Introduction

**FIGURE 1:**
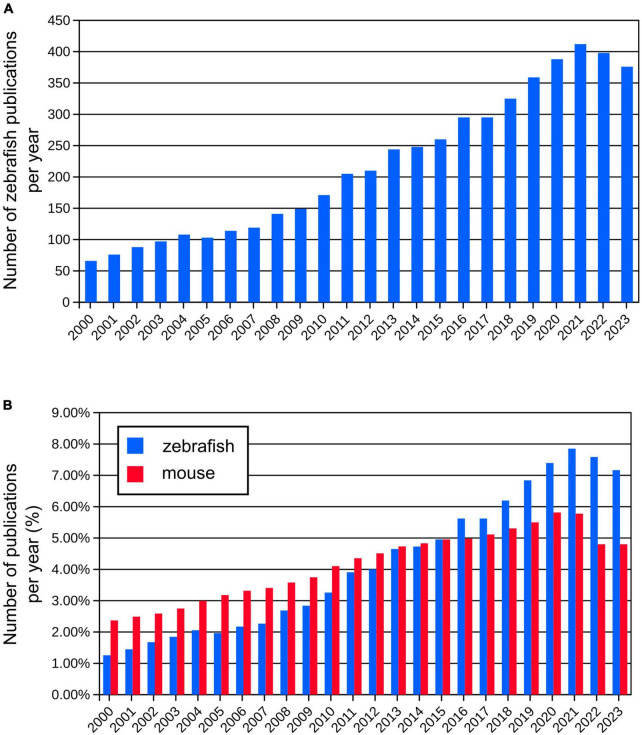
**(A)** Number of publications per year using zebrafish since year 2000 (from Pubmed database). **(B)** Comparison of relative numbers of publications per year using zebrafish and mouse models showing dynamic growth of the zebrafish research.

Since early work in the 1980s (Streisinger et al., 1981), the zebrafish (*Danio rerio*) has been increasingly used as a model organism. Although the zebrafish is a relatively new model organism for complex brain diseases, it is of great interest to scientists for a comprehensive analysis of the processes involved in the development and regeneration of the nervous system, as evidenced by the increasing number of publications using this model each year (Figure 1). The use of zebrafish in neuroscience research has several advantages. Zebrafish embryos and larvae are transparent, allowing researchers to easily visualize and study the development and function of the brain. This transparency, together with external development, makes it possible to observe the formation of neural circuits and the development of individual neurons in a living organism. Zebrafish share many genetic similarities with humans. The zebrafish genome and transcriptome have been sequenced revealing 70% sequence similarity between zebrafish and human (Howe et al., 2013; Shehwana and Konu, 2019). These features allow researchers to study fundamental processes in brain development and function that are relevant to humans (Hortopan et al., 2010; Panula et al., 2010). Also, zebrafish embryos develop quickly making them an ideal model for studying the early stages of brain development. Researchers can easily manipulate genetically and analyze the effects of genetic mutations or experimental treatments during this rapid developmental period. Zebrafish are amenable to gene knockdown, knockout, or knock-in, which allow researchers to investigate the roles of specific genes in brain development and function. These techniques – summarized in section “2 Methods to study development of the brain connectivity” – have provided valuable insights into the genetic basis of brain-related disorders. Zebrafish exhibit a range of behaviors that can be investigated providing understanding into the neural basis of various behaviors, including locomotion, learning, and social interactions (Kalueff et al., 2014). Their behaviors can be assessed using automated tracking systems, making it easier to conduct high-throughput experiments. Advanced behavioral analyses, together with neuronal activity imaging techniques are described in section “3 Methods to study brain activity and behavior” of this review. Zebrafish are relatively easy and cost-effective to maintain in a laboratory setting, which makes them an attractive model organism. Overall, zebrafish have become a valuable tool in the field of brain research because of their unique combination of characteristics that allow researchers to analyze various aspects of brain development, function, and pathology. In this review, we describe highly advanced tools for studying the brain connectivity development, brain activity, and function using zebrafish as an animal model.

## 2 Methods to study development of the brain connectivity

### 2.1 Genetic tools for selective gene expression

**FIGURE 2:**
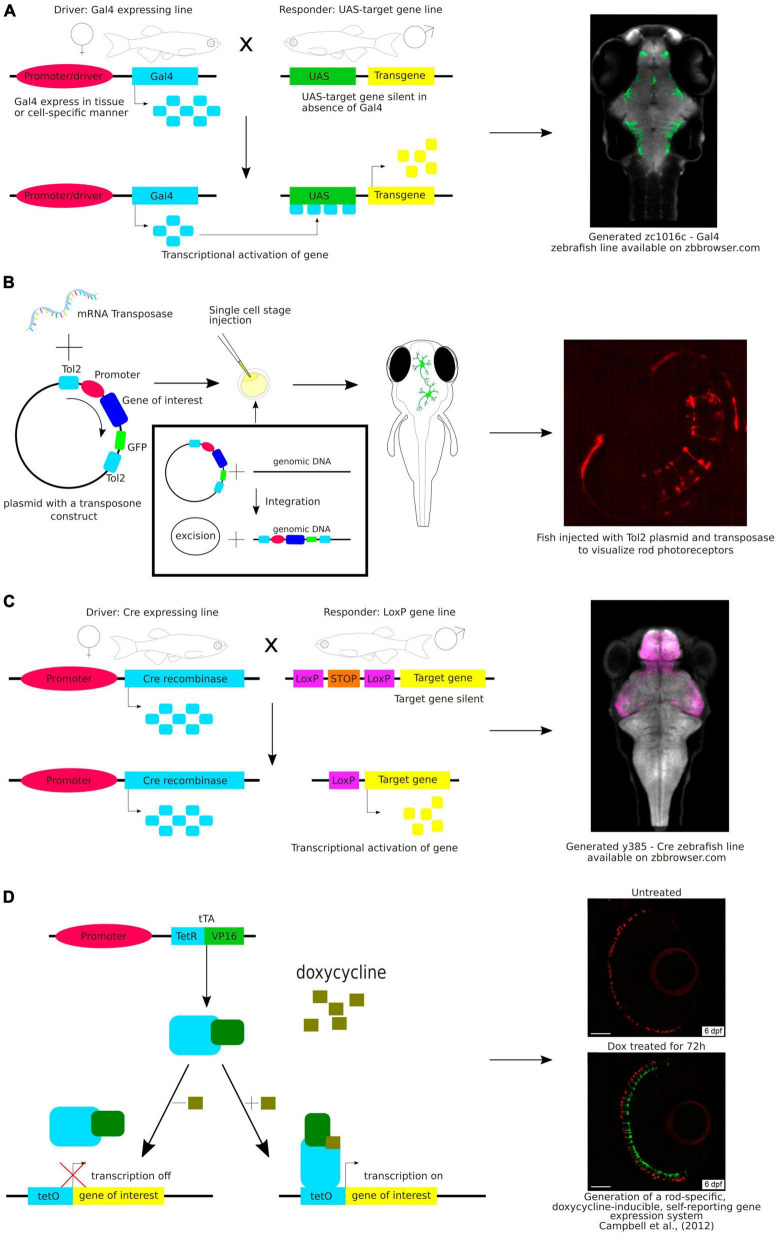
**(A)** Schematic visualization and exemplary image of the use of the Gal4-UAS system. **(B)** Schematic visualization and exemplary image of the use of the Tol2 system. **(C)** Schematic visualization and exemplary image of the use of the Cre-loxP system. **(D)** Schematic visualization and exemplary image of the use of the Tet-ON system.

Various genetic strategies are used to visualize neurons (Figure 2). One of the common approaches in molecular and cell biology is the use of strategies that require the presence of two transgenes in the same cell to activate the expression of the fluorescent reporter gene. An example of this is the galactose transcription factor (Gal4) – upstream activating sequence (UAS) system that is widely used in zebrafish research (Halpern et al., 2008). In this system, two transgenic lines are used – one with Gal4 expression in a specific region or tissue and the other with expression of the gene of interest under the UAS, e.g., green fluorescent protein (GFP). Expression of Gal4 is restricted to a specific region or to a specific tissue because it is under the control of a specific promoter. This method allows researchers to achieve highly specific and controlled gene expression patterns that enable precise manipulation and analysis of target cell populations (Figure 2A). The Gal4-UAS system is a versatile and effective tool for exploring gene functionality, observing subcellular structures, labeling specific cells or tissues, or their selective removal.

Gal4 is a yeast transcription factor consisting of a DNA-binding domain and a transcription activation domain. It binds to UAS and activates transcription from the basal promoter placed downstream of UAS. The Gal4-UAS system was first used in zebrafish by Scheer and Campos-Ortega (1999). They constructed a plasmid with the full-length yeast *GAL4* gene. However, the expression of Gal4 was weak. Since then, the method has been continuously improved. To overcome the weak expression, Köster and Fraser (2001) have used Gal4-VP16, a fusion of the DNA-binding domain of Gal4 with the transcriptional activation domain of the VP16 protein from the herpes simplex virus, which has stronger transcriptional activity. Another commonly used version of Gal4 is Gal4FF, a modified version of the Gal4 that has two activator segments of VP16 in addition to the DNA-binding domain of Gal4. Gal4FF has been shown to be better tolerated in vertebrate cells than Gal4-VP16 (Asakawa et al., 2008).

Another approach to achieve higher expression of the reporter gene was an insertion of a repeated UAS sequence in the transgene. However, Goll et al. (2009) found that the UAS sequence repeated 14 times was highly methylated, so expression of the reporter protein was silenced. Silencing and methylation were lower for UAS repeats of 5 or 4. Fish lines containing such repeats have been shown to be reliable and reproducible over more than 15 generations (Kawakami et al., 2016).

So far, many transgenic zebrafish lines expressing modified Gal4 in specific cells, organs, and tissues have been created. Several research labs have produced these Gal4 zebrafish lines in a high-throughput manner and organized them into collections available for the scientific community. These collections can be used in genetic and developmental biology research to study gene expression or manipulate gene function in a controlled and tissue-specific or temporal manner. Each Gal4 driver line in the collection is typically designed to express Gal4 in a specific tissue or cell type, e.g., *Tg(chat:Gal4)* expresses Gal4 in brain areas positive for choline acetyltransferase (ChAT) or *Tg*(*gad1b:Gal4)* with Gal4 expression in brain areas positive γ-aminobutyric acid (GABA) (Förster et al., 2017). This specificity of expression allows researchers to study development of these regions or to target them by genetic manipulations. It is important to highlight that recently the Zebrafish Brain Browser (ZBB) and its updated version, ZBB2 (Tabor et al., 2019), have become accessible online. These resources house images displaying the cellular expression patterns of various Gal4 collections, as well as transgenic lines that express fluorescent proteins in specific defined patterns (Marquart et al., 2015). This platform is particularly valuable for investigating the functional mapping of neuronal circuits. These atlases have transformed research capabilities, enabling users to easily conduct 3D spatial searches and efficiently identify lines with reporter expression in regions of interest.

In making these zebrafish transgenic lines, scientists employed the Tol2 transposon transgenesis (Figure 2B). The Tol2 system includes two key parts: a donor plasmid containing the gene of interest flanked by Tol2-transposon repeats (a Tol2 construct) and the Tol2 transposase (Kawakami, 2007). These two components are microinjected into fertilized eggs at the one-cell stage. The Tol2 construct is removed from the donor plasmid and incorporated into the genome during embryonic development. Consequently, these insertions are inherited by the subsequent fish generations. The latest generated vectors for Tol2-based transgenesis in zebrafish expand a selection of fluorescent protein choice, are coupled to Cre system or Gal4 system, and thus are adaptable for various applications. Integration of these vectors enhances the range of available tools for consistent, quality-controlled Tol2 transgenesis and gene-regulatory element testing in zebrafish and other model organisms (Kemmler et al., 2023). By using the Tol2 system combined with the Gal4-UAS, Lal et al. identified a pallial amygdala in the zebrafish brain essential for fear conditioning suggesting that it is the mammalian functional equivalent. They also provided additional bricks in our understanding of the brain evolution and diversification of neuronal networks (Lal et al., 2018).

The caveat of Tol2 transgenesis is that it uses transposon sites in the genome randomly and some of the insertions are not inherited throughout the generations. Lalonde et al. optimized a targeted vector integration system in zebrafish, by converting well-established, validated, and inherited Tol2 sites into landing sites recognized by the phiC31 integrase (Lalonde et al., 2023). They called it pIGLET, phiC31 Integrase Genomic Loci Engineered for Transgenesis. This system enables targeted insertion into a place in the zebrafish genome that is stable across generations, reducing the number of animals needed to generate transgenic lines.

Another approach to target specific neurons is the Cre-lox system, a genetic recombination system that can be used to manipulate gene expression in a cell-specific manner (Figure 2C). This system is widely used in mouse models. It also uses two transgenic lines: one that expresses the Cre recombinase enzyme in a specific subset of neurons, and another that contains a gene of interest flanked by loxP sites. When these two lines are crossed, the Cre enzyme binds to the loxP sites and e.g., excises the gene of interest resulting in a cell-specific knockout (Lin et al., 2013). Both systems, Gal4-UAS and Cre-lox, have successfully been used in zebrafish (Förster et al., 2017; Tabor et al., 2018), making this a promising strategy to achieve targeted reporter expression in neurons.

Another approach worth mentioning is the Tet-On system, an inducible gene expression system that uses tetracycline (Tet) or doxycycline (Dox) to timely control the activation of transgene expression (Figure 2D). This system has been successfully used in mammals and zebrafish (Bockamp et al., 2008; Knopf et al., 2010; Ma et al., 2017). Campbell et al. (2012) employed the Tet-On system to induce gene expression in rod photoreceptors. This proof-of-concept research shed light on the timed development and migration of these cells within the growing retina (Campbell et al., 2012).

### 2.2 Tools to study single neurons within the brain and neuronal morphology development

**FIGURE 3:**
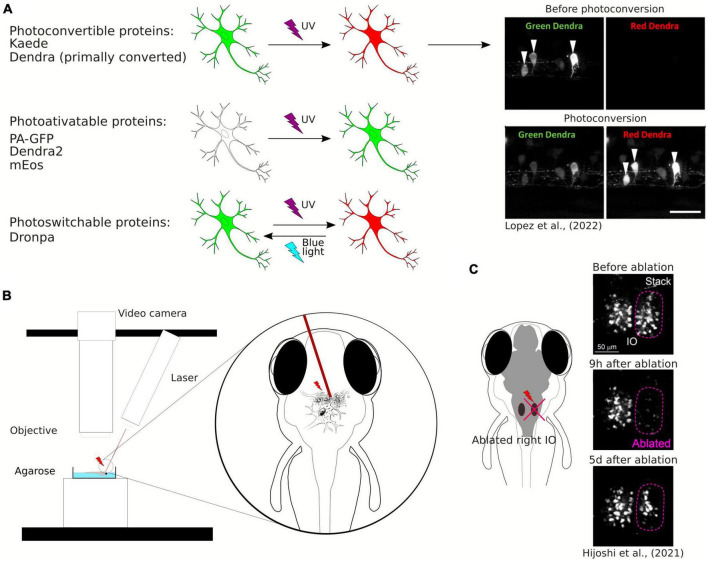
**(A)** Schematic visualization and exemplary image of the use of photoconvertible and photoactivatable proteins. **(B)** Schematic visualization of the use of the laser ablation method. **(C)** Example of the use of the laser ablation method in zebrafish brain research.

Photoconvertible and photoactivatable fluorescent proteins are widely used to study neuronal morphology, especially projecting axons and neuronal connectivity, as they allow timed visualization of single neurons within a neuronal population (Figure 3A). Photoconvertible proteins are a class of fluorescent proteins that change their emission wavelengths and thus can be activated in response to specific wavelengths of light (Chow and Vermot, 2017). They are widely used in biological research, particularly to visualize and track specific structures by fluorescence microscopy. Once activated, most photoactivatable proteins cannot be easily deactivated. On the other hand, the photoconvertible proteins emit fluorescence in one spectrum and undergo irreversible conversion to other fluorescence spectrum when exposed to violet or ultraviolet (UV) light. The first photoconvertible fluorescent protein was Kaede, and it was produced by accident. Kaede initially emits green fluorescence. When exposed to violet or UV light (350−400 nm), it undergoes irreversible conversion into a red light-emitting fluorophore (Ando et al., 2002). The use of the Kaede protein provides possibility to distinguish individual neurons from each other in the living zebrafish. In the study by Sato et al. (2006) a transgenic line *Tg(HuC:Kaede)* expressed Kaede in neurons under the neuron-specific HuC promoter and the photoconversion of Kaede enabled the labeling of a single neuron within the tectum to study development of dendritic arborization in time.

Examples of well-known photoactivatable and photoconvertible proteins include Photoactivatable Green Fluorescent Protein (PA-GFP), Dendra2, or mEos. PA-GFP is a non-fluorescent variant of GFP that can be activated by UV light and then it emits green fluorescence (Patterson and Lippincott-Schwartz, 2002). Similarly to Kaede, Dendra2 is a photoconvertible fluorescent protein converting from green to red. Dendra2 can also be photoconverted by initially exposing the protein to blue light and subsequently to near-infrared light, so called “primed conversion” (Mohr et al., 2016; Lopez et al., 2022). mEos is another photoconvertible protein that transitions from green to red fluorescence when illuminated with UV light. Another fluorescent protein discovered both with photoconvertible and photoactivatable properties is Dronpa. The interesting feature of this protein allows its fluorescence to be deactivated with blue light and reactivated with violet light (Habuchi et al., 2005). Aramaki and Hatta (2006) used Dronpa in zebrafish to visualize the development of axonal arborizations of single spinal neurons during time-lapse imaging. They photoconverted Dronpa in soma to anterogradely label the axonal terminus of a single neuron or in the axon to retrogradely label somas which the axons belong to (Aramaki and Hatta, 2006). This approach enabled to map intertwined neuronal circuitries. Along with the years, new fluorescent proteins were discovered, such as PSmOrange, with red-shifted excitation and emission wavelengths (Subach et al., 2011). Using the PSmOrange photoconversion within GFP transgenic background, Beretta et al. explored the neural circuit development of the ventral habenula by employing 2-photon microscopy time-lapse imaging. Their investigation yielded compelling evidence of the thalamic-epithalamic origin of the ventral habenula neurons (Beretta et al., 2013).

### 2.3 Remodeling of neuronal connectivity using laser ablation

One of the innovative methods used to study plasticity of neuronal connectivity in the brain is laser ablation of neuronal cells (Figure 3B). It may be also employed to study the role of a subpopulation of neurons in behavior (Muto and Kawakami, 2018) or to study single neurons connectivity development within a circuit (Hiyoshi et al., 2021). Laser ablation can be performed using a two-photon laser microscope and is especially well-suited for disrupting the activity of a limited number of specific neurons. Expression of fluorescent proteins in the specific population of neurons allows for the ablation of these neurons at desirable locations and timing at a high spatial resolution (single-cell level). Hiyoshi et al. (2021) used two-photon laser ablation of the inferior olive combined with long-term live imaging of the whole brain and showed structural remodeling of the olivo-cerebellar circuit at a single-cell resolution (Figure 3C). Irreversible ablation of neurons caused severe damage in the inferior olive and enabled live observation of remodeling of the circuit (Hiyoshi et al., 2021).

### 2.4 Tools to investigate synapse maturation live and neuronal connectivity mapping

Zebrafish have become a valuable model organism for studying synaptic structure and function. They offer several advantages for investigating synapses and neuronal connectivity mapping, including their optical transparency, rapid development, and genetic flexibility. One of the modern ways for visualizing synapses in living zebrafish larvae is the use of specialized approach such as FingRs to fluorescently label specific synaptic components. The transTANGO approach in turn, by labeling synaptically connected cells, is used to track synaptic connections. These labels can be introduced through genetic modification in a stable manner (transgenic zebrafish lines as in section “2.1 Genetic tools for selective gene expression”) or by microinjection of plasmids encoding these components. Then, the observation of synaptic dynamics in real time can be done using time-lapse imaging including synaptic plasticity and the formation and elimination of synapses during development or pathology.

**FIGURE 4:**
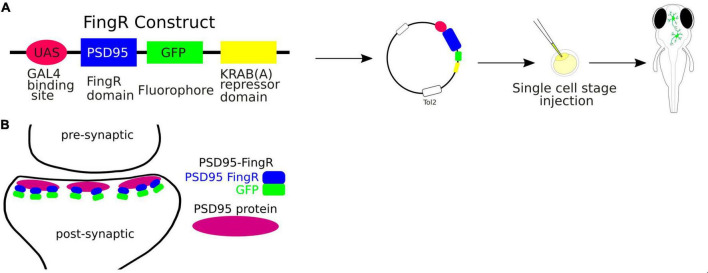
**(A)** Schematic visualization of the use of the FingRs. **(B)** FingR-PSD95 binds endogenous PSD-95 protein at the postsynaptic density.

FingRs, the fibronectin intrabodies generated by mRNA, are antibody-like proteins fused to fluorescent proteins that target endogenous synaptic proteins (Son et al., 2016; Figure 4). FingRs can be used to visualize proteins in living cells without compromising structure and function of synaptic components (Gross et al., 2013). They offer a significant advance over the conventional immunofluorescence technique, which requires cell fixation and permeabilization. Additionally, expression of FingRs is regulated by a transcriptional feedback system that utilizes a zinc-finger DNA binding domain that matches the expression level of its endogenous target (Gross et al., 2013). Thus, FingRs can be used to comprehensively map the locations, numbers, and size of synaptic connections onto specific neurons in the developing intact brain.

FingRs have been used in zebrafish to target such proteins as postsynaptic density protein 95 (PSD-95), which is localized to the excitatory synapses, or Gephyrin (GPHN), which is a component of the postsynaptic protein network of inhibitory synapses. The PSD-95 is known to interact with synaptic receptors and have been well established as a marker for the size and location of postsynaptic densities of glutamatergic synapses (Dakoji et al., 2003). Thus FingRs targeting PSD-95 allow to precisely map the excitatory synapses in terms of their sizes and locations in the living animal. Son et al. (2016) have shown that FingRs for GPHN identified sites where GABA can be uncaged in close proximity to inhibitory synapses, providing an approach for investigating inhibitory circuitry. Furthermore, the amount of GPHN at postsynaptic inhibitory sites correlated with GABA or Glycine receptors (Son et al., 2016).

**FIGURE 5:**
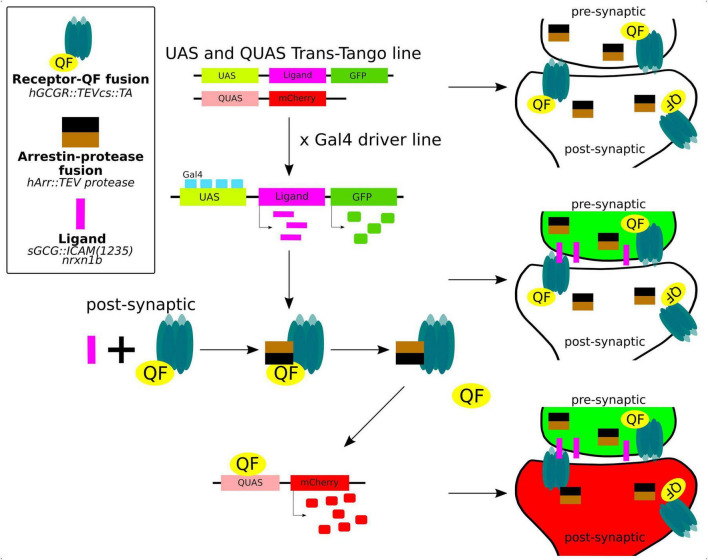
Trans-Tango method for live trans-synaptic tracing in zebrafish (details in the main text).

The trans-Tango method is a powerful and innovative technique used in neuroscience to delineate the synaptic connections between neurons in the brain (Figure 5). It is a variant of a method of identification of cells that are connected by a synapse with specific “bait” neurons, called Tango (Barnea et al., 2008). The trans-Tango method was originally developed for anterograde trans-synaptic tracing in the *Drosophilla melanogaster* olfactory system and it has been particularly useful for studying the complex neural circuits in the brain (Talay et al., 2017). In trans-Tango adopted for zebrafish, specific neurons of interest are chosen and are defined by expression of ligand that serves as a “bait” to identify their synaptic partners. Ligand expression is controlled by UAS, and thus by a Gal4 driver line of interest. The ligand is derived from human glucagon (sGCG), is tethered to the presynaptic membrane through the transmembrane domain of zebrafish Nrxn1b, and extended into the synaptic cleft through the linker sequence (Talay et al., 2017). All neurons in the central nervous system are genetically modified to express (1) a G protein coupled receptor (GPCR) fused to QF transcription factor on their cell surface, and (2) a fusion of TEV protease and Arrestin. GPCR-QF fusion is regulated by TEV protease because the TEV cleavage site lies in between the two proteins. In presynaptic neurons, Gal4 binds to UAS resulting in the expression of the sGCG ligand and its presentation on the presynaptic membrane. The presynaptically-presented sGCG ligand binds to the GPCR-QF fusion protein on the postsynaptic membrane, causing recruitment of Arrestin-TEV and cleavage of QF. QF then translocates to the nucleus and promotes transcription of a fluorescent marker under QUAS. This marker helps identify the cells that are synaptically connected to the bait neuron. The advantage of this method is that it works with any neural circuit regardless of the neurotransmitter that it uses (Talay et al., 2017; Coomer et al., 2023).

### 2.5 Fluorescence Resonance Energy Transfer (FRET) to study protein interactions or conformation in the living brain

**FIGURE 6:**
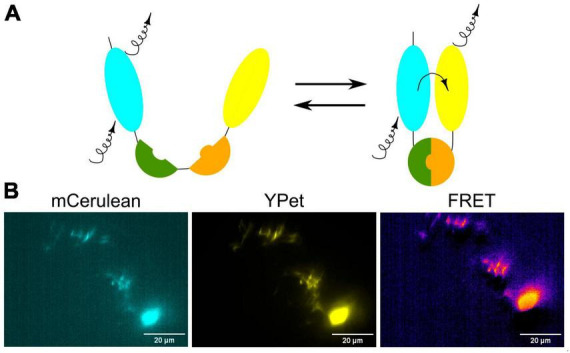
**(A)** Schematic visualization of the FRET principle. **(B)** Exemplary image of neurons in the zebrafish brain showing FRET.

Fluorescence Resonance Energy Transfer (FRET) is a powerful and widely used technique in molecular and cellular biology that allows researchers to study interactions and conformational changes between molecules, typically within the nanometer scale. FRET relies on the transfer of energy between two fluorescent molecules when they are in close proximity (Figures 6A, B). This transfer happens from a donor fluorophore to an acceptor fluorophore. In the context of zebrafish research, FRET can be used to investigate various biological processes and molecular interactions, including interactions between proteins or within a protein in the living zebrafish (Kardash et al., 2011). By genetically fusing a donor fluorophore to one protein and an acceptor fluorophore to another, researchers can measure the proximity of these proteins and infer whether they are interacting in specific cellular contexts. To detect conformational changes in proteins, the donor and acceptor fluorophores are attached to different parts of a protein of interest and the changes in protein shape or structure in response to various stimuli or biological processes can be monitored by FRET in the living zebrafish neurons (Figure 6B).

## 3 Methods to study brain activity and behavior

### 3.1 Tools for single-cell neuronal activity analysis in the zebrafish brain

Neuronal activity causes rapid changes in intracellular free calcium. Using this mechanism, genetically encoded calcium indicators (GECIs) allow for real-time, *in vivo*, non-invasive measurement of neuronal activity, on a spatial scale ranging from synapses (Chen et al., 2013) to large populations of neurons (Ahrens et al., 2013). Currently, GFP-based GECIs are most widely used (Chen et al., 2013). The original GCaMP probe consisted of circularly permuted green fluorescent protein (cpGFP), the calcium-binding protein calmodulin (CaM), and CaM-interacting M13 peptide (Nakai et al., 2001). The major principle of GCaMP action is based on conformational changes in CaM/M13 upon calcium ion binding, resulting in increased brightness of the green fluorescence (Chen et al., 2013; Dana et al., 2016). Since the discovery of GCaMP, many variants of this fusion protein have been generated and widely used in many *in vitro* and *in vivo* models. For example, the GCaMP5G reporter (Ahrens et al., 2013) was used by us to examine epilepsy in the zebrafish model of Tuberous Sclerosis Complex. Whole-brain imaging of *tsc2*^*vu*242^;*Tg(GCaMP5G)* larvae with light-sheet microscopy has revealed hyperexcitability in the pallial neurons of the *tsc2*^*vu*242/*vu*242^ mutant fish (Kedra et al., 2020).

Subsequent iterations of GCaMP calcium indicators have evolved from the original structure to enhance sensitivity and response kinetics. While the precise biophysical mechanisms governing these improvements are not yet fully elucidated, a trade-off appears to exist between the sensitivity and kinetics of GCaMP sensors. Consequently, distinct variants have been developed, optimized either for heightened sensitivity (e.g., GCaMP6s, GCaMP7s) or accelerated response kinetics (e.g., GCaMP6f and GCaMP7f) (Chen et al., 2013; Dana et al., 2019). Furthermore, large-scale neuronal activity imaging often encounters challenges related to signal cross-talk stemming from white matter regions and the nuclear expression of GECIs limit the imaging speed (Kim et al., 2014). Addressing this, Shemesh et al. (2020) have engineered cell body-targeted GCaMP6f and GCaMP7f which exhibited superior signal-to-noise ratios and reduced false-positive correlations when employed in the imaging of dense neural circuits. More recently, Zhang et al. have introduced the 8th generation of GCaMP indicators, denoted as jGCaMP8 sensors. These sensors offer rapid response kinetics, along with heightened sensitivity compared to preceding GECIs. jGCaMP8 variants are available, each optimized for either sensitivity or speed (Zhang et al., 2023). jGCaMP8s has recently been used as the calcium reporter in a newly developed platform that combines Ca^2+^ imaging and optogenetics in freely moving zebrafish (Chai et al., 2024). SyjGCaMP8m, a variant of GCaMP8 expressed in synaptic terminals, has been used to image bipolar cells in the inner retina of larval zebrafish, in order to investigate the role of amacrine and bipolar cells in the processing of color information (Wang et al., 2023).

Notwithstanding the sensitivity and signal-to-noise ratio offered by GFP-based GECIs, their utility is restrained by the characteristics of their excitation and emission spectra (Table 1). Notably, these indicators are incompatible with transgenic organisms that already express GFP-based proteins. Also, the excitation spectrum of GCaMP partially overlaps with that of rhodopsin-based ion channels frequently employed in optogenetic investigations. To circumvent these constraints, the adoption of red GECIs has emerged as a solution. Red GECIs not only alleviate these issues but also exhibit diminished susceptibility to light scattering and absorption within tissues, along with reduced phototoxicity. Dana et al. have devised two enhanced red GECIs based on mRuby (jRCaMP1a and jRCaMP1b) and mApple (jRGECO1a). Beyond their application in cultured rat hippocampal neurons, mice, *Drosophilla*, *C. elegans*, and zebrafish, where they were pan-neuronally expressed under the HuC promoter, these red GECIs have displayed striking advantages over previously available RFP-based sensors. They have exhibited sensitivities similar to GCaMP6 and have maintained stable performance over time. Particularly jRGECO1a has shown superior sensitivity compared to jRCaMP1, representing an advance in the detection of neural activity relative to prior generations of red GECIs. Notably, jRCaMP1a and jRCaMP1b have demonstrated resistance to photoswitching following blue light exposure, a contrast to jRGECO1a, which undergoes such a conversion (Dana et al., 2016). The concurrent utilization of both green and red GECIs was demonstrated by Mu et al. (2019) in a study featuring zebrafish expressing GCaMP6f in neurons and jRGECO1b in radial astrocytes. This dual application facilitated the separate but simultaneous imaging of the two distinct cell types, thereby elucidating the dynamic interplay between neuronal and glial activity in the modulation of motor and passive states (Mu et al., 2019).

**TABLE 1:** Tools for visualization of the neuronal activity live.

Name	Targeted mechanism	Fluorophore	Excitation/emission wavelengths	Utility	References
GCaMP5G/6/7/8	Ca^2+^ influx	cpGFP**	488/507	Visualizing neuronal activity dynamics	Ahrens et al., 2013 Kedra et al., 2020 Shemesh et al., 2020 Zhang et al., 2023
jRCaMP1a/1b	Ca^2+^ influx	mRuby	558/605	Visualizing neuronal activity dynamics; can be used together with GFP-based sensor for labeling distinct cell populations	Dana et al., 2016
jRGECO1a	Ca^2+^ influx	mApple	568/592		
CaMPARI2	Ca^2+^ influx	EosFP*	Green: 502/516 Red: 562/577	Marking of active neuronal populations by irreversible photoconversion	Moeyaert et al., 2018
Ratiometric cameleons	Ca^2+^ influx	Two fluorophores that allow FRET to occur between them		Visualizing neuronal activity dynamics compare to the basal levels of Ca^2+^	Fan et al., 2007 Tao et al., 2011
Voltron/Voltron2	Membrane potential	Janelia Fluor_525/549/552/585_	JF_525_: 525/549 JF_549_: 549/571 JF_552_: 552/575 JF_585_: 585/609	Detection of action potentials and subthreshold voltage signals	Abdelfattah et al., 2019 Abdelfattah et al., 2023
ASAP1	Membrane potential	cpGFP	488/507		St-Pierre et al., 2014 Silic et al., 2022
zArchon1	Membrane potential	GFP	488/507		Piatkevich et al., 2018 Böhm et al., 2022
GRAB_*DA*_	Dopamine release and uptake	cpGFP	488/507	Visualizing dopaminergic signaling	Sun et al., 2018
GRAB_*NE*_	Norepinephrine release and uptake	cpGFP	488/507	Visualizing norepinephrinergic signaling	Feng et al., 2019
GRAB_5–HT1_._0_	Serotonin release and uptake	cpGFP	488/507	Visualizing serotonergic signaling	Wan et al., 2021
iGluSnFR	Glutamate release and uptake	cpGFP	488/507	Visualizing glutamatergic signaling	Marvin et al., 2013

*FP, fluorescent protein;

**cpGFP, circularly permuted GFP.

A slightly different approach is offered by the genetically encoded indicator CaMPARI, the second generation of which has been released recently (Fosque et al., 2015; Moeyaert et al., 2018). CaMPARI is a photoconvertible green fluorescent protein that, under UV light exposure, undergoes irreversible photoconversion to a red fluorescent form, but only in the presence of free calcium ions. Importantly, the degree of red-to-green fluorescence is contingent upon the levels of calcium allowing for the precise capture of neuronal activity in response to specific stimuli, both *in vivo* and *in vitro*. While CaMPARI probes have not been as extensively used as GCaMPs, they have proven to be valuable tool for the screens aimed at assessing the neurotoxicity of environmental contaminants or pharmaceuticals (Kanyo et al., 2021; Biechele-Speziale et al., 2023). Recently, live imaging of zebrafish larvae expressing CaMPARI was instrumental in elucidating the mechanism of Kv7 voltage-gated potassium channel inhibition (Kanyo et al., 2023).

Another approach to visualize and measure calcium dynamics upon neuronal activity is to use FRET-based calcium indicators called cameleons. Similarly to GECIs, cameleons are genetically encoded Ca^2+^ indicators based on fluorescent proteins and CaM (Fan et al., 2007; Tsuruwaka et al., 2007). Cameleons are especially valuable for assessing basal-level activity and have found application in functional mapping and connectivity mapping studies in zebrafish (Tao et al., 2011). There are currently several different cameleons classified by composition of their Ca^2+^ binding domains. One of the most widely used cameleon is Yellow cameleon that consists of two fluorescent proteins, cyan CFP and yellow YFP. The neural activity can be visualized by FRET between these fluorescent proteins (Hasani et al., 2023). This technique has been used to observe Ca^2+^ dynamics in sensory and spinal cord neurons in zebrafish (Higashijima et al., 2003).

Cameleons typically use two fluorophores, a donor and an acceptor, which can be engineered to emit different wavelengths of light upon calcium binding and they can be used in combination with other FRET-based sensors targeting different molecules (e.g., cAMP and pH) to simultaneously monitor multiple cellular processes within the same cell. Moreover, cameleons provide ratiometric measurements comparing the emission of the donor and acceptor fluorophores, which reduces the impact of factors like indicator concentration and illumination intensity, resulting in more quantitative and accurate calcium measurements. FRET-based indicators often exhibit a higher signal-to-noise ratio compared to some single-fluorophore indicators like GCaMP probes. This means that cameleons can provide more robust and reliable measurements, especially in challenging experimental conditions. They can be designed to have a wider dynamic range, allowing them to detect both small and large changes in calcium concentrations. However, it’s essential to note that cameleons can be more technically challenging to implement than the single-fluorophore indicators, as they often require more complex microscopy setups. The choice between FRET-based indicators and other approaches will depend on the specific experimental goals, the availability of equipment, and the expertise of the researchers. Both types of indicators have their strengths and are valuable tools in the field of calcium imaging and zebrafish research.

In parallel with the ongoing efforts to improve the kinetics and sensitivity of calcium indicators, researchers have explored genetically encoded voltage indicators (GEVIs; Table 1). These indicators are based on naturally occurring ion channel voltage sensor domains fused to fluorescent proteins or, in later iterations, microbial rhodopsin proteins [reviewed in (Xu et al., 2017)]. Voltage imaging is technically challenging, necessitating fast imaging speeds without compromising brightness or sensitivity. One innovative approach, proposed by Abdelfattah et al., involves the creation of a hybrid “chemigenetic” voltage indicator named Voltron. This indicator combines a voltage-sensitive microbial rhodopsin domain with a dye-capture protein domain, allowing for the binding of synthetic fluorophores such as Janelia Fluor. In this approach, transmembrane voltage-dependent changes in the rhodopsin domain modulate the fluorescence quenching of the dye molecule through FRET. Voltron excels in terms of brightness and photostability when compared to previous GEVIs. In zebrafish experiments, Voltron has been utilized to detect action potentials and subthreshold voltage signals during swim bouts in response to visual cues, providing insights into sensorimotor integration at an unprecedented temporal resolution (Abdelfattah et al., 2019). Recently, Abdelfattah et al. (2023) have also developed an improved version of this indicator, Voltron2, optimized for higher sensitivity to action potentials, subthreshold potential fluctuations, and it was tested *in vivo* in zebrafish olfactory neurons.

An alternative approach to monitor neuronal activity *in vivo* is tracking of neurotransmitter release. For example, a genetically encoded G-protein-coupled receptor-based dopamine sensor (GRAB_*DA*_) was engineered by coupling a conformationally sensitive cpGFP to a selected human dopamine receptor. It allows for rapid, sensitive, and specific detection of extracellular dopamine enabling quantitation of dopamine release in the living animals. GRAB_*DA*_ was successfully used in zebrafish larvae to observe dopamine dynamics in response to a threatening looming stimulus (Sun et al., 2018). A year later, an analogous norepinephrine sensor was developed by Feng et al. (2019), and similarly validated in zebrafish larvae, also by observing the response to a looming stimulus. Both sensors showed high specificity, sensitivity, brightness, photostability, and lack of phototoxicity (Sun et al., 2018; Feng et al., 2019). Using a similar strategy, Wan et al. have developed the GRAB_5–HT1_._0_ sensor for detection of serotonin (Wan et al., 2021), and while it has not yet been used in zebrafish studies, it likely could be adapted for zebrafish similarly to its predecessors.

Moreover, iGluSnFR is a glutamate reporter constructed from *E. coli* GltI, and cpGFP, originally shown to reliably report glutamate release from excitatory synapses in zebrafish (Marvin et al., 2013; James et al., 2019). It has been recently improved by postsynaptic targeting, achieved by the fusion of iGluSnFR with glutamate receptor auxiliary proteins γ-2 and γ-8, creating the SnFR-γ2 and SnFR-γ8 reporters (Hao et al., 2023). However, they have not yet been tested in zebrafish. For differential properties of various approaches to study brain activity see Table 1.

### 3.2 The virtual reality for the analysis of behavior

Virtual Reality (VR) is an emerging tool to study zebrafish behavior. VR arenas, displaying 2D or 3D environments, can be programmed to provide realistic visual cues and feedback, serving to analyze how the fish interact with their surroundings. This technology enables the execution of assays wherein the ongoing simulation is dynamically adjusted to the fish’s behavior. This capability facilitates the observation of intricate, purpose-driven behaviors, including goal-directed navigation, hunting, or social interactions, all within the limits of a comparatively confined physical setting. The VR configuration effectively mitigates the spatial constraints associated with traditional behavioral tests. The mobile projection can persist for variable duration and at diverse speeds, and it can follow any trajectory. Additionally, it allows for simulating any desired visual stimuli, ranging from simple shapes and patterns to complex environments.

The simplest VR assays involve the projection of an animated virtual object on the walls of the tank. Zebrafish react to a moving dot stimulus as if it were a potential predator or prey depending on the dot size (Barker and Baier, 2015). Since this discovery, multiple studies have used such stimuli to examine the mechanisms of prey tracking (Trivedi and Bollmann, 2013; Barker and Baier, 2015; Jouary et al., 2016; Antinucci et al., 2019). More recently, researchers have also used the VR projection of conspecifics for the analysis of shoaling, and how the presence and behavior of conspecifics affects decision making in both freely swimming (Harpaz et al., 2021; Oscar et al., 2023) as well as head-restrained zebrafish (Huang et al., 2020). In the case of larvae, it has been demonstrated that a simple shape corresponding in size and movement patterns to a real zebrafish larva, is sufficient to provoke the same social responses as live conspecifics (Harpaz et al., 2021). However, in the case of adults, Huang et al. (2020) have shown that the fish are able to differentiate between a simple shape and a more detailed projection, and require a more realistic image of an adult zebrafish to trigger social behavior. And while the currently available technology, based on rendering the VR environment from the perspective of a single animal, does not allow for simultaneous testing of multiple fish in a single arena, virtual projections of conspecifics can be sufficiently sophisticated and accurate to the real-world behavior that they have been used to create models of sensorimotor control during schooling in juvenile zebrafish. In addition to studying social behavior in zebrafish, these models were used to refine the movement of autonomous vehicles, showing that insights gained from zebrafish behavioral studies can find application outside the field of neuroscience, or even biology (Li et al., 2023).

Another simple type of a VR assay, developed by Portugues and Engert (2011) is based on displaying a 2D drifting striped pattern to create the impression of movement. Presented with the moving stimulus, the fish responds by tail movements corresponding to the behavior observed during free swimming, and the visual feedback can be controlled by the researcher to create a closed-loop environment (Portugues and Engert, 2011). In a more complex application of this method, Torigoe et al. (2021) have created a 3D arena with moving patterns of various colors. By pairing different projections with the absence or presence of an electric shock, they were able to induce a positive or negative associations with the displayed stimuli, and demonstrated how the fish use this information to choose a safe environment and learn new information when the visual cues were changed (Torigoe et al., 2021).

Researchers have harnessed the advanced capabilities of VR tools to conduct innovative experiments. For instance, the FreemoVR system developed by Stowers et al. (2017) enables “virtual teleportation,” wherein fish experience a seamless transition to a new projected environment as they move within a designated region. 3D VR environments such as FreemoVR are usually created using open-source software for game developers (Stowers et al., 2017; Huang et al., 2020). However, such tools might require optimization to make them suitable for behavioral research. BonVision, created by Lopes et al. (2021) is an open-source software package for creating and displaying 2D and 3D VR environments, compatible with various display devices, equipped with a library of predefined 3D structures, and capable of real-time control and automatic calibration of the virtual environment using deep neural networks. BonZeb, similarly based on the open-source visual programming language Bonsai, is a specialized set of modular software packages designed for high-resolution zebrafish behavioral tracking, both in head-fixed and free-swimming virtual assays (Guilbeault et al., 2021). VR environments offer immense potential for behavioral research, with tools like BonVision and BonZeb facilitating the generation of 2D and 3D VR environments, real-time control, and precise calibration, all tailored for zebrafish studies.

### 3.3 Calcium imaging coupled to behavior to study brain function

A distinct advantage of VR over traditional experimental setups is the possibility of simulating a changing, moving environment while the fish remains fixed in place, allowing for simultaneous microscopic imaging of brain activity together with behavioral assay. A fictive swimming assay, developed by Masino and Fetcho (2005) for the analysis of spinal motor patterns using electrophysiological recordings was adapted for a behavioral test in VR, where immobilized zebrafish larvae are placed inside a water-filled chamber fitted with screens for projecting stimuli (Mu et al., 2019). Alternatively, specific parts of the larval body, such as the tail and eyes, may be freed from agarose while still maintaining immobilization (Trivedi and Bollmann, 2013; Jouary et al., 2016). When studying behaviors that necessitate the use of adult fish, which cannot be easily immobilized in agarose, methods include head fixation (Huang et al., 2020) or the use of custom-made harnesses (Torigoe et al., 2021). In fictive swimming experiments, the firing of motor neurons in the tail is monitored to quantify movement speed and direction, enabling precise visual feedback (Masino and Fetcho, 2005; Ahrens et al., 2012; Mu et al., 2019). In instances where the fish’s tail remains mobile, swimming patterns can be predicted based on tail curvature (Huang et al., 2020) or extrapolated from tail movements recorded during previous free-swimming periods (Jouary et al., 2016). Unlike traditional setups, VR permits the withholding or modification of visual stimuli, enabling the investigation of the fish’s responses to these alterations. For instance, Mu et al. (2019) explored how fish respond to repeated futile movements and adapt their behavioral strategy in response to failure. The presented visual pattern was abruptly halted while the fish continued to “move” (Mu et al., 2019). In a study by Yang et al. (2022) which investigated the mechanisms of self-location and positional homeostasis, artificial movement was played in reverse, creating the illusion of struggling against a water current. This allowed an examination of how zebrafish react to perceived displacement and maintain their position (Yang et al., 2022). These studies, elucidating neuronal circuits underpinning specific motor behaviors, exemplify the capacity of VR to facilitate *in vivo* brain imaging during simulated movements.

VR experiments involving fictive swimming allow for *in vivo*, real time monitoring of neuronal activity during a behavioral test. For this, two-photon microscopy is the most suitable providing robust 3D resolution with minimal photo-damage. Additionally, the infrared light used for excitation should not elicit visual responses in zebrafish (Renninger and Orger, 2013), and therefore should not interfere with the behavioral assay. Vladimirov et al. (2014) have developed an alternative approach for neuronal activity imaging during fictive swimming. This setup was based on lightsheet microscopy in which one laser beam scanned the brain from the front, imaging the forebrain between the eyes. At the same time, the second beam scanned the fish from the side, switching off when it passed over the eye, thus avoiding direct stimulation of the retina that would confound the results of the behavioral test (Vladimirov et al., 2014). Other groups have also constructed behavioral testing chambers coupled with imaging systems built to suit their particular needs. Vanwalleghem et al. (2020) placed immobilized zebrafish larvae in a microfluidic device designed to deliver water flow stimuli, and imaged them on a custom-built light-sheet microscope. Constantin et al. (2020) also utilized a custom-built light-sheet microscope to measure neuronal activity in response to visual and auditory stimuli. While the smaller larval brain is more accessible for imaging, two-photon microscopy was also used to perform calcium imaging during behavioral tests in adult fish (Huang et al., 2020; Torigoe et al., 2021). Huang et al. (2020) were able to visualize individual somata and neurites up to a depth of >200 μm below the brain surface .

However, the methods described above require restraining the fish, which limits the range of behaviors that can be studied or might result in unnatural movements. Immobilizing the head causes reactive forces in the body that do not naturally occur during free swimming, and restricts the movement to a horizontal plane, causing aberrant vestibular feedback. The CaMPARI calcium indicator (described in section “3.1 Tools for single-cell neuronal activity analysis in the zebrafish brain”) allows for irreversible marking of active cells during free swimming (Fosque et al., 2015). Furthermore, Kim et al. (2017) have devised a sophisticated tracking microscope system that incorporates a motorized stage with a motion-cancellation mechanism that cancels brain motion in three dimensions enabling brain imaging in freely-swimming fish. The motion cancellation is coupled with a differential illumination focal filtering (DIFF) microscopy approach, based on an earlier technique called HiLo microscopy, described by Lim et al. (2011). HiLo microscopy used uniform and spatially modulated illumination in a speckled or grid pattern and combined these raw images resulting in an image of higher resolution. In DIFF microscopy, the images are acquired with a pair of complementary grid illumination patterns (Kim et al., 2017). This integrated system facilitates the continuous real-time imaging of neural activity in the brain of freely swimming larval zebrafish for extended periods.

### 3.4 Optogenetic approaches coupled to behavioral analysis

**FIGURE 7:**
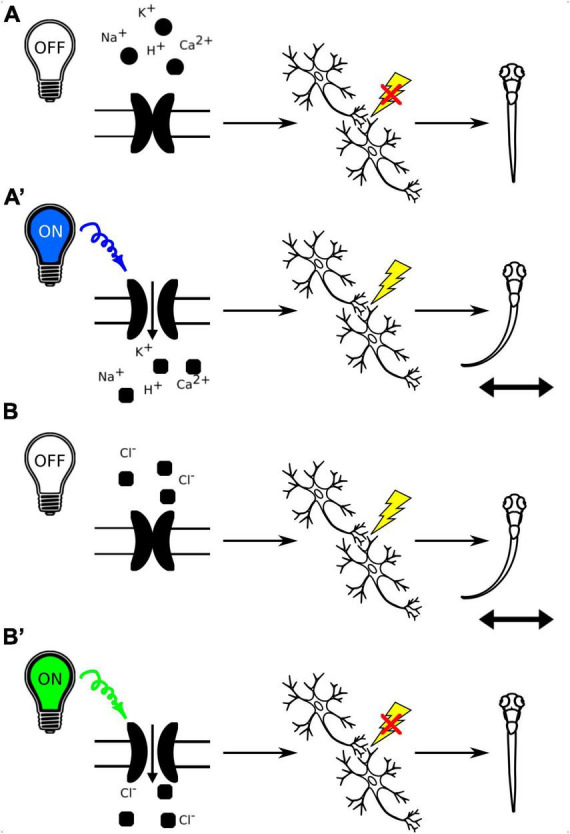
**(A)** Schematic representation of the use of channelrhodopsins to activate neuronal circuits with light-off **(A)** and light-on conditions **(A’)**. B. Schematic representation of the use of anion channelrhodopsins to inactivate neuronal circuits with light-off **(B)** and light-on conditions **(B’)**.

Optogenetic tools allow for specific and reversible manipulation of activity of selected neurons in a living organism, enabling researchers to experimentally test the link between neuronal activity and behavioral responses (Figure 7). Moreover, the transparent zebrafish larvae allow for non-invasive optogenetic modulation of neural activity throughout the brain (Portugues et al., 2013). The cation channelrhodopsins (ChR1 and ChR2), originally found in the green alga *Chlamydomonas reinhardtii*, are among the most popular optogenetic tools. They are light-activated cation channels that allow the passage of positively charged ions upon light activation. They consist of rhodopsin, a seven-transmembrane helix protein with covalently linked retinal, acting as the chromophore. Upon illumination with light of specific wavelength, they generate action potentials in normally light-insensitive neurons which result in activation of the neuronal circuit (Nagel et al., 2003; Berthold et al., 2008). Anion channelrhodopsins are a type of optogenetic tool derived from microbial rhodopsins that are able to inactivate neuronal circuit. When exposed to specific light spectrum, anion channelrhodopsins open and enable the influx of negatively charged ions, primarily chloride ions, into the target neurons, leading to hyperpolarization of the cell membrane and suppression of neuronal activity (Mohamed et al., 2017). By expressing anion channelrhodopsins in specific neurons and then illuminating them with the appropriate light, researchers can inhibit neuronal firing and investigate the functional role of these neurons in various physiological and behavioral processes in a precise and temporally controlled manner. Both cation and anion channelrhodopsins have become valuable tools for studying neural circuits and their functions in neuroscience research.

A toolkit of nine transgenic zebrafish lines expressing optogenetic actuators under UAS – CoChR, CheRiff, ChR2_(*H*134*R*)_, eArch3.0, GtACR1, GtACR2, Chronos, ChrimsonR and eNpHR3.0 – was generated by Antinucci et al. (2020), and tested using high-throughput behavioral assays and *in vivo* whole-cell electrophysiological recordings, providing a resource for design and calibration of opsin-expressing zebrafish lines in specific regions. These lines utilize the Gal4-UAS system for targeted opsin expression, and provide stable, reproducible opsin expression levels across cells and generations. Optogenetic interrogation of simple behaviors are done with modified microscopes or automated tracking systems that include LED lights (Oikonomou et al., 2019; Antinucci et al., 2020). Concurrently, the Raspberry Pi Virtual Reality (PiVR) system was purpose-built for the optogenetic stimulation of unrestrained animals to study more complex behaviors. The PiVR platform offers the capability to present virtual environments while automating behavioral tracking and facilitating feedback based on the animal’s behavioral responses. Optogenetic manipulations are achieved through the use of a LED light source that remains focused on the animal during its locomotion. Although this setup has been utilized in phototaxis experiments with zebrafish larvae, it’s important to note that to date the optogenetic component has been exclusively tested in *Drosophilla* (Tadres and Louis, 2020).

In zebrafish, the photoactivation of ChR2 in somatosensory neurons or ear hair cells has been demonstrated to elicit escape responses, as documented in multiple studies (Douglass et al., 2008; Monesson-Olson et al., 2014; Ozdemir et al., 2021). Notably, optogenetic manipulation using ChR2 was also employed in Barker and Baier’s research concerning responses to moving dot stimuli, revealing that the activation of a specific subset of glutamatergic tectal interneurons prompted an increased approach toward small-sized objects mimicking prey (Barker and Baier, 2015). Recent investigations have further harnessed ChR2 to unveil two distinct modules of hypothalamic dopaminergic neurons in zebrafish. These modules were found to play pivotal roles in the initiation of locomotor activity, responsiveness to acoustic stimuli, and the impact of olfactory cues on decision-making processes (Barrios et al., 2020).

GtACR1 and GtACR2 represent anion channelrhodopsins derived from the alga *Guillardia theta*. These optogenetic tools have demonstrated their efficacy in inhibiting spontaneous coiling movements in larval zebrafish, underscoring their utility for the optical modulation of behavior in zebrafish larvae (Mohamed et al., 2017). However, it is important to acknowledge the limitations of GtACRs. Activation of these pumps in cells with elevated intracellular chloride levels may result in depolarization. Also, they can induce neuronal depolarization upon light cessation, potentially causing a burst of neural activity. Additionally, there is uncertainty regarding their sustained functionality beyond a one-minute time frame. Consequently, GtACRs are primarily suited for the observation of acute processes confined to the duration of light application (Mohamed et al., 2017). Nonetheless, as optogenetic inhibitors, GtACRs can serve as valuable controls alongside cation channelrhodopsins. For instance, in a study by Cheng et al. (2017) the identification of a nucleus in the anterior thalamus that responds to light stimuli, subsequently evoking a response in the habenula, was initially achieved by stimulating this nucleus with ChR2. This elicited depolarization in the habenula and an increase in neuronal activity, as measured by GCaMP6f fluorescence. Conversely, the application of GtACR1 inhibited the light response and disrupted phototaxic behavior, thereby confirming the nucleus’s role in regulating habenula function (Cheng et al., 2017).

Another approach to inhibit neuronal activity is the PAC-K silencer, based on photoactivated adenylyl cyclases (PACs) and the small cyclic nucleotide-gated potassium channel SthK. PAC-K acts as a light-controlled K^+^ channel, allowing for controlled hyperpolarization of neurons (Bernal Sierra et al., 2018). One advantage of the PAC-K silencer is that it is sensitive to longer wavelengths of light compared to GtACRs. This can be beneficial because longer wavelengths of light penetrate tissues more effectively, allowing for deeper and more precise light activation *in vivo*. Also, the PAC-K silencer typically requires lower light intensities for activation compared to GtACRs. This can be less phototoxic and reduce the risk of heating in the tissue, which is important for long-term experiments or when studying sensitive neural circuits. GtACRs and other anion channelrhodopsins are sensitive to intracellular chloride levels. If a cell has high intracellular chloride concentrations, activating GtACR may lead to depolarization rather than hyperpolarization. PAC-K is less affected by intracellular chloride levels, making it a more reliable tool in diverse cellular contexts (Bernal Sierra et al., 2018). Anion channelrhodopsins like GtACR can sometimes cause depolarization of neurons when the light is turned off. PAC-K may exhibit a reduced off-response, making it more suitable for experiments, where the precise timing of inhibition is crucial. PAC-K can also be activated for a longer duration compared to GtACRs, which may be advantageous for experiments that require sustained inhibition of neural activity. The choice between these optogenetic tools depends on the specific experimental requirements and the characteristics of the neural circuit being studied.

Additionally, the combination of optogenetic circuit interrogation and calcium imaging has been realized through the innovative work of dal Maschio et al. (2017). They developed a comprehensive protocol employing both ChR2 and GCaMP6s to facilitate concurrent 3D two-photon optogenetic stimulation and calcium imaging. This study harnessed two-photon computer-generated holography for photostimulation, leveraging a spatial light modulator to project intricate illumination patterns. Additionally, the co-expression of photoactivatable PA-GFP, ChR2, and GCaMP6s enabled the labeling and visualization of cells of interest, providing insights into their morphology subsequent to functional characterization. This methodology was instrumental in the identification of neuronal ensembles in the tegmentum of larval zebrafish associated with the control of tail bending (dal Maschio et al., 2017). Until now, such experiments have usually been conducted on head-restrained zebrafish, and optogenetic manipulation in freely swimming fish required full-field illumination, and in consequence the generation of fish lines expressing the photosensitive proteins in the selected brain region. However, Chai et al. have recently developed a system for targeted brain imaging and optogenetics in freely-moving fish. The use of light-field microscopy allows for rapid recording of neuronal activity. In addition to the GFP-based Ca^2+^ indicator, the fish also expressed an activity-independent RFP, serving as a reference signal. By using this reference channel alongside a machine learning-based image detection and registration algorithm, researchers were able to correct Ca^2+^ signal artifacts caused by zebrafish movements. The tracking system was also used to deliver photostimulation to the region of interest previously chosen in the software, based on the ZBB atlas data (Chai et al., 2024). This approach allows to study zebrafish brain activity during more naturalistic behavior than possible in experimental setups requiring the restraining of fish.

### 3.5 *In vivo* electrophysiological studies of the zebrafish brain

The easily accessible zebrafish brain also allows for electrophysiological recordings. In particular, multiple zebrafish models of epilepsy are used, in which the presence of seizures is confirmed by recording of electrical discharges in the brain. Epileptic seizures in zebrafish were first shown by Baraban using extracellular field recording. A microelectrode was inserted several microns into the forebrain of larval zebrafish, and the electrical activity of the brain was recorded, showing seizure-like discharges following the administration of convulsant drugs, or in genetically modified fish carrying a mutation in the *tsc1a* gene, associated with the disease Tuberous Sclerosis Complex, the symptoms of which include epilepsy (Baraban, 2013). Later, Eimon et al. (2018) have developed a platform for simultaneous recording of local field potentials (LFP) in multiple fish, and have used it to analyze brain activity in the zebrafish model of a different genetic disease resulting in epilepsy, Dravet syndrome. More recently, LFP recording has been combined with Ca^2+^ imaging in order to map epileptiform activity in the brains of *kcnj10a* morphant zebrafish as well as fish treated with the proconvulsant drug pentylentetrazole (PTZ), and evaluate the efficacy of the anti-epileptic drug valproate in these models (Cozzolino et al., 2020). In the meantime, Pinion et al. (2022) have analyzed and catalogued electrographic signatures of LFPs in zebrafish treated with different pro-convulsant agents, and compared these recordings with the results of electrophysiological studies in mammals, further illustrating the translational potential of zebrafish models for studies of epilepsy.

The utility of electrophysiological studies in zebrafish is not, however, limited to studying epilepsy. In the previously mentioned study by Masino and Fetcho (2005), foundational to establishing the paradigm of fictive behavior, whole cell patch clamp recordings were taken to measure the activity of motor neurons during the behavioral test. Intracellular recordings of the Mauthner neurons, responsible for integration of sensory information and evoking a motor response, were used to test the precise effect of commonly used anesthetics on different aspects of sensory processing, providing with a method to evaluate potential novel anesthetic compounds (Machnik et al., 2018). Vestibulospinal neurons were targeted in voltage- and current-clamp recordings aimed to elucidate how the larval zebrafish utilize vestibulospinal input to maintain posture. The use of these methods revealed the role of both inhibitory and excitatory inputs in posture stabilization, demonstrating the utility of zebrafish in studying the function of neuronal circuits, particularly when, in contrast to mammals, they are more accessible for *in vivo* patch clamp recording (Hamling et al., 2023).

Hong et al. (2016) have developed a system for non-invasive, long-term electrophysiological recording of zebrafish, through the use of a microfluidic system. The so-called Zebrafish Analysis Platform (iZAP) involves a central chamber where multiple zebrafish larvae can swim freely, and from there enter single channels where they become restrained. There, multiple electrodes are positioned in contact with the head, and field potentials from the dorsal side of the zebrafish forebrain, optic tectum, midbrain and hindbrain are measured. This system was successfully validated through recording of PTZ-induced seizures in zebrafish larvae, and allowed for nearly continuous 130 h recording of brain activity – a length of recording not possible with previously established, more invasive techniques, requiring immobilization in agarose and penetrating electrodes. The iZAP system also allows for changing of the medium without disturbing the larvae, making it useful for high-throughput drug testing (Hong et al., 2016). More recently, Tomasello and Sive (2020) have also developed a simple method of measuring brain and spinal cord activity in larval zebrafish, based on a previously available microelectrode array from Axion Biosystems. This system was validated by collecting LFP recordings from zebrafish larvae at 7 dpf, with the anticonvulsant drug valproic acid used as a positive control for lowered brain activity. While this method does involve immobilizing the fish in agarose, and therefore cannot be used for such extended periods of time as the iZAP method, it is a useful tool for non-invasive electrophysiological recordings in live zebrafish, making use of an easy to use, commercially available technology (Tomasello and Sive, 2020).

## 4 Discussion

While zebrafish are a valuable and versatile model organism for brain research, they do have certain limitations that researchers should consider when designing experiments and interpreting results. Zebrafish have a less complex brain compared to mammals. Some areas of the brain found in mammals are absent or simplified in zebrafish. This means that findings in zebrafish may not always directly translate to humans. Zebrafish behaviors are less complex than those of humans, which can make it challenging to model complex behaviors and cognitive functions in zebrafish. Zebrafish do not possess language or consciousness, which makes it challenging to study brain functions related to these aspects of cognition and behavior. While there is genetic homology between zebrafish and humans, there are also significant differences in the genetic makeup. This can limit the extent to which zebrafish findings can be extrapolated to mammals. Also, the small size of zebrafish can be a limitation when conducting invasive experiments or when detailed anatomical or electrophysiological measurements are required. Zebrafish have a relatively short lifespan compared to some other model organisms, which can be a limitation when studying long-term processes.

While genetic tools for manipulating the zebrafish genome have advanced significantly, they may not be as extensive as those available for other model organisms like mice. Zebrafish research is still relatively young compared to research in mice and rats. This means that there may be gaps in our knowledge, and some areas of zebrafish neuroscience are still emerging. Despite these limitations, zebrafish remain a valuable tool in brain research, particularly in areas related to early brain development, neural circuitry, and certain neurological and neuropsychiatric conditions. One example of a success story in zebrafish research is the discovery of an anti-seizure medication called clemizole. In a drug-repurposing study that tested the approved medications in zebrafish model of pediatric epilepsy, Dravet syndrome, clemizole was identified as a potent drug with anti-seizure actions with the use of high-throughput behavioral tests and validation by electrophysiological recordings (Baraban et al., 2013). This discovery was a foundation of clinical trials testing the safety and efficacy of clemizole as a treatment for Dravet syndrome, of which phase II is to be completed by the end of 2024 (NCT04069689, NCT04462770, ClinicalTrials.gov). Zebrafish are often used in combination with other model organisms to provide a more comprehensive understanding of brain function and dysfunction. Researchers should carefully consider these limitations when using zebrafish and design experiments to address specific questions that can be effectively tackled using this model organism.

## Author contributions

OD: Investigation, Visualization, Writing – original draft, Writing – review & editing. TD: Investigation, Visualization, Writing – original draft, Writing – review & editing. JZ: Conceptualization, Formal Analysis, Funding acquisition, Investigation, Supervision, Visualization, Writing – original draft, Writing – review & editing.
